# Formation of massive iron deposits linked to explosive volcanic eruptions

**DOI:** 10.1038/s41598-018-33206-3

**Published:** 2018-10-05

**Authors:** J. Tomás Ovalle, Nikita L. La Cruz, Martin Reich, Fernando Barra, Adam C. Simon, Brian A. Konecke, María A. Rodriguez-Mustafa, Artur P. Deditius, Tristan M. Childress, Diego Morata

**Affiliations:** 1 Department of Geology and Andean Geothermal Center of Excellence (CEGA), FCFM, Universidad de Chile, Plaza Ercilla 803, Santiago, Chile; 2 Department of Earth and Environmental Sciences, University of Michigan, 1100 North University Avenue, Ann Arbor, MI 48109-1005 USA; 3 School of Engineering and Information Technology, Murdoch University, Perth, WA 6150 Australia

## Abstract

The genetic link between magmas and ore deposit formation is well documented by studies of fossil hydrothermal systems associated with magmatic intrusions at depth. However, the role of explosive volcanic processes as active agents of mineralization remains unexplored owing to the fact that metals and volatiles are released into the atmosphere during the eruption of arc volcanoes. Here, we draw on observations of the uniquely preserved El Laco iron deposit in the Central Andes to shed new light on the metallogenic role of explosive volcanism that operates on a global scale. The massive magnetite (Fe_3_O_4_) ore bodies at El Laco have surface structures remarkably similar to basaltic lava flows, stimulating controversy about their origin. A long-standing debate has endured because all proposed models were constructed based exclusively on samples collected from surface outcrops representing the uppermost and most altered portion of the deposit. We overcome this sampling bias by studying samples retrieved from several drill cores and surface outcrops. Our results reveal complex lithological, textural and geochemical variations characterized by magmatic-like features and, most notably, a systematic increase in titanium concentration of magnetite with depth that account for an evolving system transitioning from purely magmatic to magmatic-hydrothermal conditions. We conclude that El Laco, and similar deposits worldwide, formed by a synergistic combination of common magmatic processes enhanced during the evolution of caldera-related explosive volcanic systems.

## Introduction

Ore deposits are normally formed by magmatic-hydrothermal processes over a range of depths within the upper crust. However, the role of subaerial volcanic processes in metallogenesis is poorly understood. In fact, volcanic eruptions preclude the formation of mineral deposits near the surface because metals and volatiles are vented during explosive events^1^. Here, we provide new evidence that supports subaerial volcanic eruptions in magmatic arcs as a viable process for the accumulation of metals at or near the surface by focusing on the world class El Laco iron deposit in the Central Andes.

**Figure 1:**
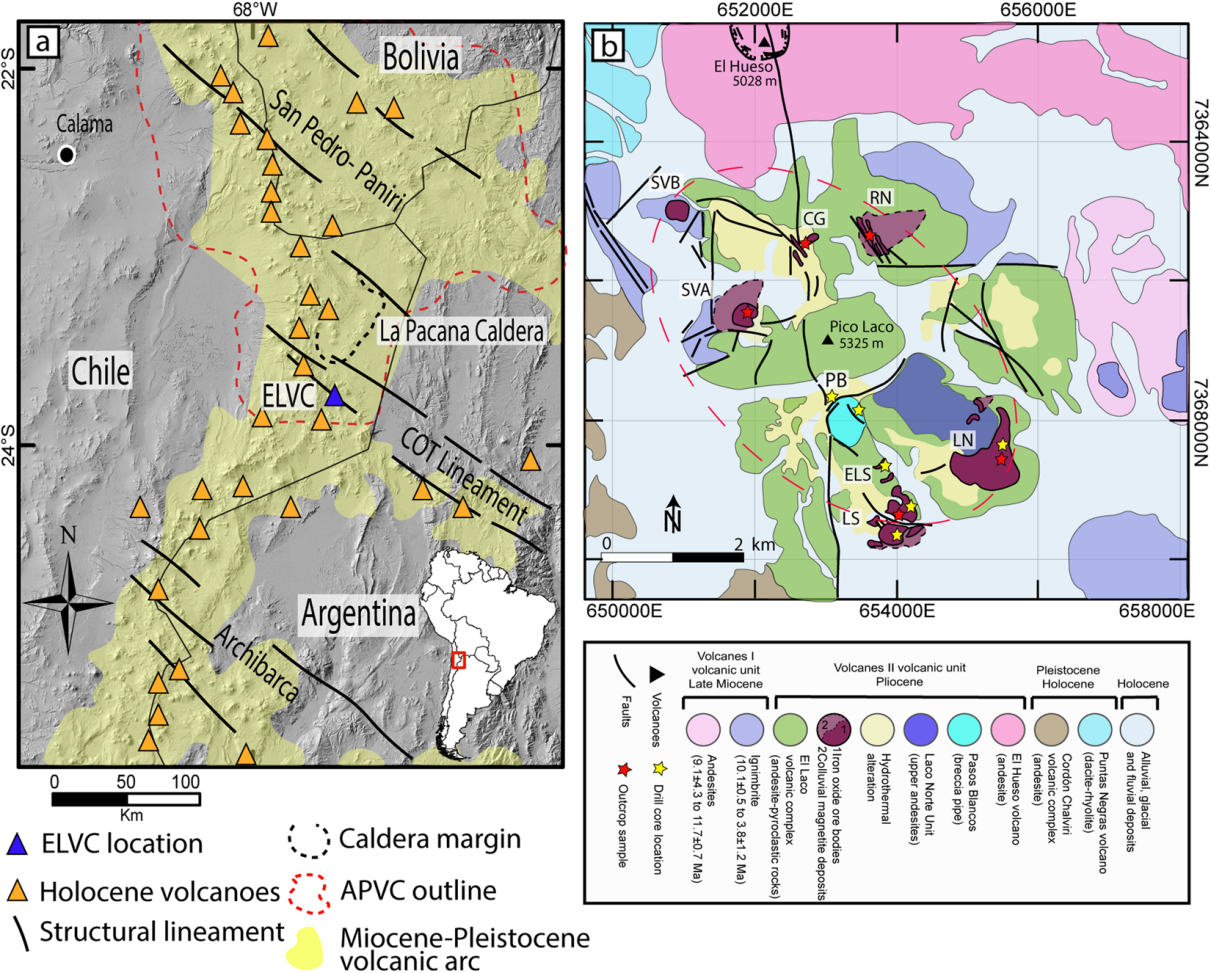
(**a**) Location of the ELVC within the main Miocene-Pleistocene volcanic arc (yellow area). The Altiplano-Puna Volcanic Complex (dashed red line) and the NW-trending structural lineaments are also shown. (**b**) Geologic map of the El Laco Volcanic Complex showing the location of the studied drill cores and surface samples. Based on mapping by the Compañía Minera del Pacífico (CAP Minería). LN: Laco Norte, LS: Laco Sur, ELS: Extensión Laco Sur, PB: Pasos Blancos, SVA: San Vicente Alto, SVB: San Vicente Bajo, CG: Cristales Grandes, RN: Rodados Negros. The NW-trending spatial distribution of the magnetite ore bodies is illustrated by the dashed red ellipse.

The El Laco volcanic complex (ELVC) hosts world class magnetite (Fe_3_O_4_) deposits with remarkable volcanic and subvolcanic features. The ELVC, formed on an exceptionally thick crust (58–76 km)^2^, is located at the southeast margin of one of the Earth’s most extensive volcanic plateaus, built during the late Miocene by an ignimbrite flare-up (Altiplano-Puna volcanic complex, APVC)^3^. In addition, it is spatially associated with the NW–SE trending Calama–Olacapato–El Toro (COT) lineament, which is responsible for the alignment of Neogene-Quaternary volcanic activity in the region^4^ (Fig. 1a). The ELVC is the product of a complex volcanic history developed from the Miocene to Pleistocene, punctuated by several volcanic events including explosive eruptions leading to stratovolcano collapse, resurgent volcanic activity, fissural emissions and late stages of intense hydrothermal activity^5–7^. These events resulted in a cluster of andesitic to dacitic volcanic structures comprising numerous NW-trending fissural emission centers and secondary craters associated with ring-shaped structures around Pico Laco. These volcanic products range in age from 5.3 ± 1.9 to 1.6 ± 0.5 Ma^7^, and the structures mark the position of a collapsed ancient crater, whose resurgent magmatic activity formed Pico Laco^5^.

The world-class El Laco iron deposit in the ELVC consists of six massive magnetite ore bodies that are spatially associated with the pre-existing subvertical annular collapse structures and secondary craters that form a NW-trending ellipse around Pico Laco^5,7^ (Fig. 1b). Total estimated resources are 733.9 Mt at an average ore grade of 49.2% Fe^8^. The ore bodies are interbedded between andesitic to dacitic lava flows and pyroclastic rocks, displaying different morphologies such as lava-like flows (Laco Norte, Laco Sur, and San Vicente Alto), NW-trending tabular bodies (Rodados Negros and Cristales Grandes) and dome-shaped bodies (San Vicente Bajo)^9^. The ore bodies are largely composed of magnetite with minor diopside, scapolite, apatite, REE-rich and iron phosphates, and hematite-goethite alteration formed by supergene oxidation of original magnetite.

## Depth-dependent textures and geochemical gradients of magnetite

**Figure 2:**
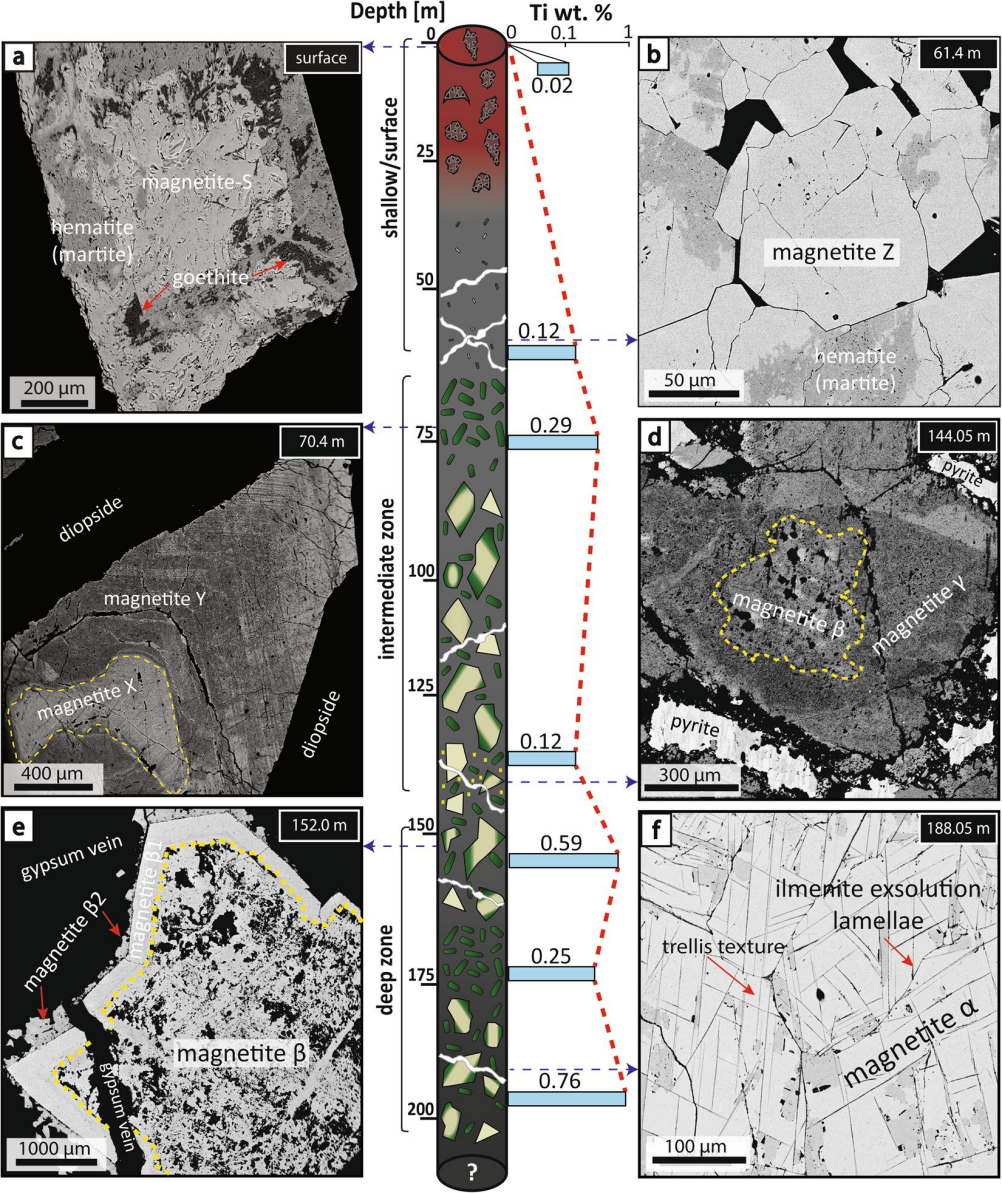
Schematic illustration of a representative drill core from Laco Norte (LCN-0944) showing the morphology and lithological variations of the ore bodies. The dashed red line and light blue bars indicate the average Ti concentration of the magnetite from the surface to the base of the drill core. Backscattered electron (BSE) images show the different textural types of magnetite. (**a**) Euhedral, altered and porous Magnetite-S grain from a surface sample showing pervasive replacement by hematite and goethite along grain boundaries, fractures, and pores. (**b**) Aggregate of pristine Magnetite-Z grains, weakly oxidized to hematite (sample depth 61 m). (**c**) Microcrystalline core of pristine Magnetite-X showing evidence of dissolution-reprecipitation processes. Magnetite-Y overgrowths Magnetite-X, and exhibits alternating bands of Ti-bearing inclusions, which are responsible for the increase in the magnetite Ti concentration within this zone (sample depth 70.4 m). (**d**) Ti-poor Magnetite-γ from pyrite-rich massive magnetite zone associated with gypsum veinlets. Magnetite-γ overgrowth developed on an inclusion-rich Magnetite-β core and displays sector zoning with inclusion-rich areas (sample depth 144 m). (**e**) Euhedral core of inclusion-rich Magnetite-β surrounded by a pristine inclusion-free-rim of Magnetite-β1. Both magnetite types have high Ti contents. Irregular grains of Ti-poor Magnetite-β2 associated with late crosscutting gypsum veinlets developed over Magnetite-β1 rims (sample depth: 152 m). (**f**) Aggregate of Ti-rich Magnetite-α grains showing well-developed ilmenite exsolution lamellae, which exhibit both trellis and sandwich textures (sample depth 188.05 m).

**Figure 3:**
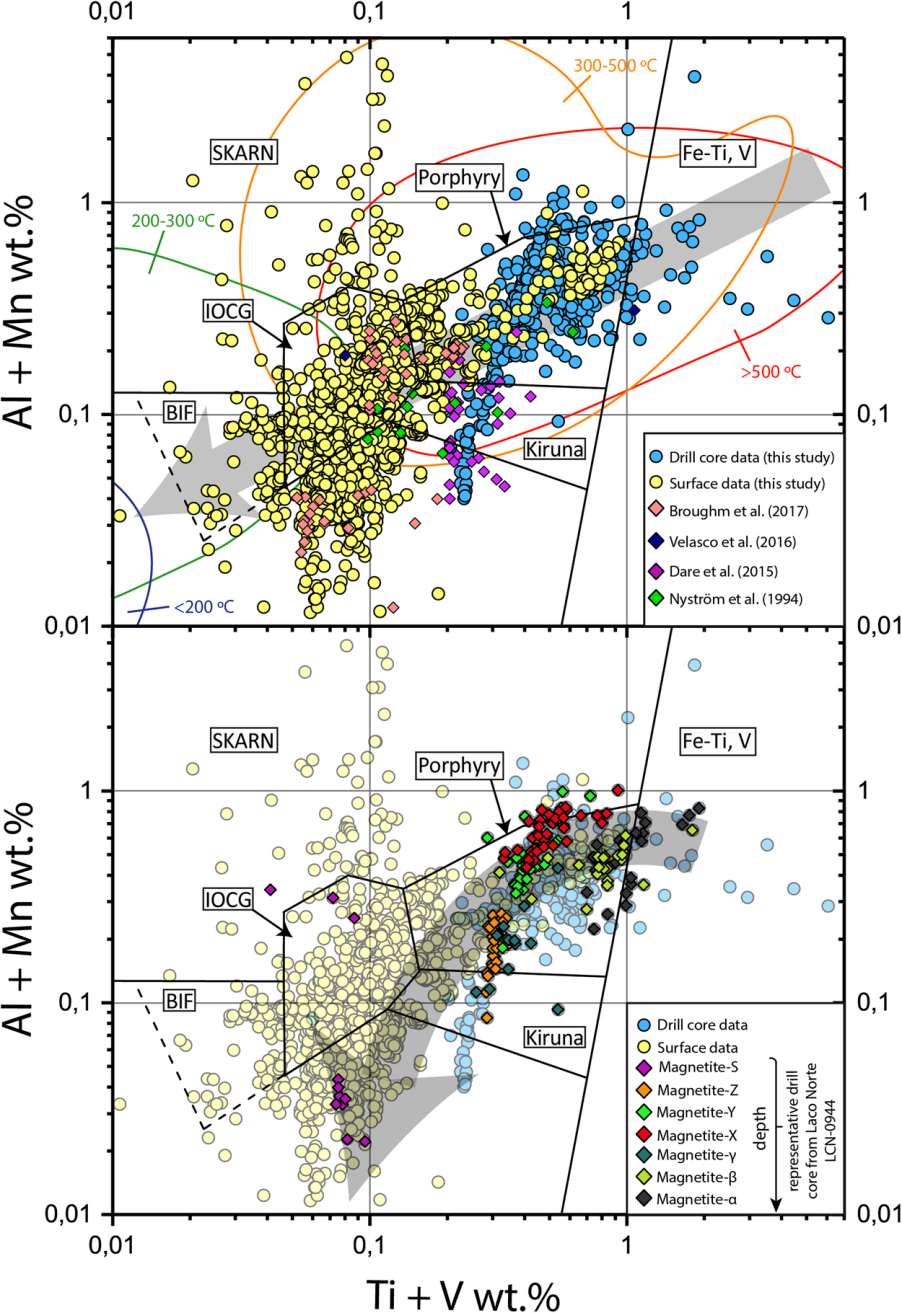
(**a**,**b**) [Al + Mn] versus [Ti + V] plots (in wt. %) showing the chemical variations of magnetite from El Laco. (**a**) The plot shows all EPMA data for magnetite from both drill core samples (blue circles) and surface outcrops (yellow circles) (Supp. Mat. Appendix_1). The colored diamonds correspond to magnetite data from surface samples reported in previous studies. The colored contours are from ref.^11^ and show estimated formation temperatures for the deposits. Magnetite from most drill core samples plot towards the high-temperature fields (*Fe-Ti, V; Porphyry* and *Kiruna fields*), whereas data points from surface samples show a larger dispersion, plotting both in the high, moderate and low temperature fields (*Porphyry, Skarn, IOCG, Kiruna* and *BIF fields*). This indicates a transitional cooling trend from purely magmatic conditions to magmatic-hydrothermal conditions from depth to surface, which is represented by the straight grey arrow. (**b**) The plot shows EPMA data points from the representative Laco Norte drill core in Fig. 2, illustrating the compositional variability at the grain scale from depth (Magnetite-α) to surface (Magnetite-S); note that data from previously published studies are not included in (**b**). The curved grey arrow represents a typical cooling trend.

Investigation of surface and drill core samples revealed a complex lithological, textural and geochemical zoning for magnetite from the Laco Norte, Laco Sur, and Extensión Laco Sur ore bodies (Fig. 1b and Supp. Mat. Figs S1–S3). All magnetite ore bodies have a similar structure with massive magnetite characterized by lava-like textures up to intermediate depths (~80–90 m), followed by magnetite breccias that can extend to depths below 200 m (Fig. 2 and Supp. Mat. Figs S1–S4). Based on lithological and mineralogical characteristics, the Laco Norte ore body was divided into three main zones: shallow/surface, intermediate, and deep zones (Fig. 2). Within these zones, different types of magnetite were identified based on micro-textural and geochemical criteria. EPMA analyses reveal significant surface-to-depth variations in magnetite composition (Figs 2 and 3a,b; Supp. Mat. Appendix_1). Particularly, Ti, V, Al, and Mn concentrations in magnetite increase progressively with depth (Fig. 3b).

The shallow/surface zone (0–65 m) comprises the main part of the massive magnetite body, including its outcropping portion (Fig. 2a and Supp. Mat. Figs S1–S4). Mineralogically, it is composed of >90% modal magnetite with minor diopside and scarce apatite. The zone displays a vesicular texture, which is more common at shallower levels. Euhedral magnetite crystals cover the walls of large vesicles. Magnetite in the outcropping body is coarse-grained (Magnetite-S, >500 µm size) and shows a high degree of alteration to hematite and goethite (Fig. 2a). Iron phosphates and inclusions of monazite and thorium silicate occur in magnetite from the upper levels, particularly at grain rims, or filling both fractures and vesicles. BSE images show that Magnetite-S is characterized by abundant microporosity and pervasive replacement by hematite and goethite mostly along grain boundaries, fractures, and pores (Fig. 2a). These magnetite grains display a large compositional variability with average concentrations for Mg and Si of 7522 and 4650 ppm, respectively, and low V (586 ppm) and Ti (218 ppm) contents.

At depths between ~30–65 m, the Fe-oxide mineralogy is dominated by Magnetite-Z, which is a weakly to moderately oxidized magnetite characterized by hematite formation along rims or through fractures (Fig. 2b). Late gypsum veinlets crosscut and fill open spaces in massive Magnetite-Z down to ~45 m. At depths between 40 and 65 m, Magnetite-Z forms aggregates of pristine, mostly euhedral crystals, ranging in size from ~10 to 120 µm. Magnetite-Z has a relatively high Mg content (average 7203 ppm) and is depleted in Si, Ca and Al (Appendix 1, Supp. Material); Ti and V concentrations average 1236 and 1861 ppm, respectively.

The intermediate zone (~65–145 m) is characterized by a transition from the shallow massive magnetite to a magnetite breccia body (Fig. 2). A massive magnetite level is present at ~65 m, which contains abundant coarse-grained euhedral diopside grains that vary in size from a few tens of micrometers to a few millimeters, immersed in a texturally diverse magnetite matrix (Fig. 2c). Magnetite in this diopside-rich zone displays two distinct textures. Magnetite-X occurs as pristine microcrystalline cores with grain sizes approximately 500 µm. It is replaced by Magnetite-Y via dissolution-reprecipitation processes (Fig. 2c). Magnetite-Y overgrowths Magnetite-X and is characterized by an oscillatory texture, i.e., alternating inclusion-rich (trace element enriched) and inclusion-poor (trace element depleted) bands (Fig. 2c). The thickness of the individual bands varies between ~4 to 100 µm, whereas the inclusion sizes vary from 10′s to 100′s of nanometers to a few micrometers. This leads to significant compositional variability between Magnetite-X and -Y, about a few wt. % for Si and Mg, and 1000′s ppm for Al, Ti, and Ca. Titanium-bearing silicate inclusions in Magnetite-Y increase the Ti concentration of these grains, reaching average contents of up to 3380 ppm, while Magnetite-X has lower Ti contents (2359 ppm). However, the average concentration of V is similar in both types (1802 and 1803 ppm for Magnetite-X and Magnetite-Y, respectively). A pyrite-rich magnetite zone associated with late gypsum veinlets occurs at 144 m depth. It is dominated by magnetite grains (Magnetite-γ) formed as overgrowths on inclusion-rich relict cores of magnetite-β (Fig. 2d). Magnetite-γ has a low Ti (average = 1232 ppm) and high V (average = 2547 ppm) concentrations.

The deep zone (~150–200 m) comprises a breccia body with andesite fragments replaced by fine-grained diopside within a magnetite-diopside matrix. Two types of magnetite grains are identified at different depths: Magnetite-β (152.0 m; Fig. 2e) and Magnetite-α (188.05 m; Fig. 2f). Both have similar compositions, but different textures. Magnetite-β occurs commonly as coarse-grained euhedral crystals that contain inclusion-rich cores surrounded by inclusion-free rims (Magnetite-β1; Fig. 2e). A third (late) generation of smaller anhedral magnetite crystals (Magnetite-β2) associated with gypsum veinlets occurs at the outer rim of the inclusion-free magnetite (Fig. 2e). The Ti and V concentrations for Magnetite-β are high (average of 6144 and 2578 ppm, respectively), and similar to Magnetite-β1 (average Ti = 5970 ppm; V = 2583 ppm). In contrast, Magnetite- β2 has the lowest Ti and V contents (610 and 100 ppm, respectively). Magnetite-α forms an aggregate of titanomagnetite crystals, ~100–300 µm in size, with well-developed ilmenite exsolution lamellae and oxidation zones along grain rims and in fractures (Fig. 2f). EPMA data revealed that Magnetite-α contains the highest average contents of Ti and V at El Laco (7637 and 2631 ppm, respectively).

The trace element geochemistry of magnetite and its genetic interpretation have been extensively explored in a variety of mineralized systems^10,11^. These studies have used compositional differences of Al, Mn, Ti, and V in magnetite to construct discrimination diagrams that are used to differentiate between various styles of mineralization. Magnetite from surface and drill core samples at El Laco forms two distinct populations in a [Al + Mn] versus [Ti + V] diagram^11^ (Fig. 3a). This plot illustrates the distinctive trend from high-temperature magnetite that progressively grades towards lower-temperature, hydrothermal compositions. The drill core data (blue circles) show a distinctive trend from igneous signatures at depth, to hydrothermal compositions towards the surface. The majority of the drill core data plot within the *Porphyry* field, which comprises magnetite crystallized from moderate- to high-temperature magmatic-hydrothermal fluids. Magnetite from the deepest drill core samples from Laco Norte and Laco Sur plot within the magmatic *Fe-Ti, V* field; i.e., their chemistry is consistent with that for magnetite that crystallized from a silicate melt^11^. On the other hand, surface samples (yellow circles) show a large dispersion, plotting in almost all fields except the magmatic field. Most surface data points plot towards low [Ti + V]; i.e., lower temperature conditions, particularly within the *IOCG-*, *BIF* fields, and even beyond the boundaries for these fields (Fig. 3a). These compositions are characteristic of growing magnetite from moderate to low-T hydrothermal fluids or magnetite that has been chemically re-equilibrated by lower temperature fluids after mineralization^11^.

Figure 3b depicts the geochemical signature of the different magnetite generations identified at different depths in the drill core samples. Consistently, the deep zone magnetite grains from both Laco Norte (e.g., Magnetite-α and -β, Fig. 2e,f) and Laco Sur, extend from the purely magmatic *Fe-Ti, V* field to the *Porphyry* field (Fig. 3b). These magnetites have the highest content of Ti (up to 5.86 wt. % and average of 7082 ppm), V (up to 3900 ppm and average of 2246 ppm) and Al (up to 8200 ppm and average of 3746 ppm). The magmatic affinity of Magnetite-α is further confirmed by the well-developed ilmenite exsolution lamellae observed in these grains (Fig. 2f), which are typical of Ti-rich magnetite or titanomagnetite in magmatic Fe-Ti oxide deposits and accessory Fe-Ti oxides in igneous rocks^12–14^. Magnetite grains from intermediate depths, i.e., Magnetite-X and -Y, plot in the upper part of *Porphyry* field (Fig. 3b). Magnetite-X and -Y are more depleted in Ti and V relative to the early-crystallized Magnetite-α and -β, and configure a descending temperature trend that suggests crystallization from an evolving magmatic-hydrothermal aqueous fluid at high-temperature conditions; i.e., >500 °C^11^. Chemical variations and micro-textural relationships between Magnetite-X and -Y indicate that dissolution-reprecipitation processes played an important role during magnetite growth^15,16^, resulting in precipitation of Magnetite-Y with crystallographically-controlled alternating inclusion-rich/inclusion-poor bands from a fluctuating composition hydrothermal fluid^17^, leading to an increase in the trace element concentrations in certain zones (arrow in Fig. 3b).

Magnetite grains from the upper zones are depleted in trace elements and pristine when compared with magnetite from deeper levels. They plot along the decreasing temperature trend from the lower part of the *Porphyry* field to *Kiruna* field (Fig. 3b). It is likely that Magnetite-Z has chemically equilibrated with the Fe-rich ore fluid, as evidenced by the lack of detectable porosity (Fig. 2b)^15^, and homogeneous distribution of lower amounts of Al, Si, Ca, Ti and V in comparison with magnetite types -α, -β, -X and -Y; these observations are consistent with published data for surface samples^18^. Magnetite-S, representative of the Laco Norte surface samples, marks the end of the cooling trend (Fig. 3b), and its geochemical signature is consistent with published data^19^.

## The optimal pathways for iron enrichment

**Figure 4:**
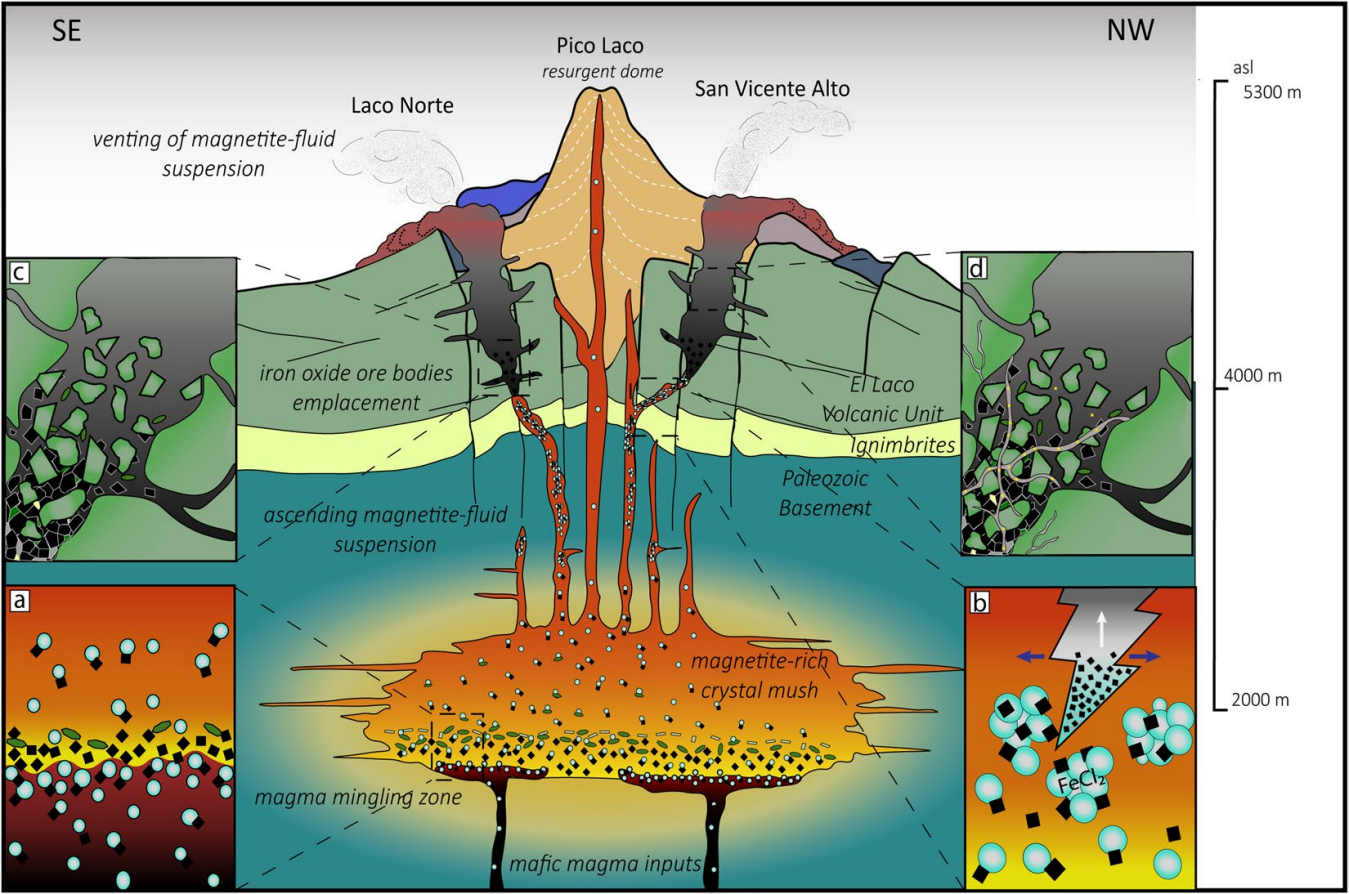
Proposed genetic model for the El Laco iron deposit. (**a**) Formation of a settled magnetite-rich crystal mush in an andesitic magma chamber below the ELVC. Water-saturated magnetite-bearing mafic magma underplates the andesitic magma body; exsolved bubbles nucleate on magnetite crystals from the mafic magma. Bubbles rise to form a magnetite + bubbles-rich foam at the interface, which can be transferred to the overlying magma chamber. Once the supersaturation pressure is reached, large populations of bubbles of supercritical fluid are exsolved from the andesitic reservoir, which along with the bubbles transferred by magma mingling processes, preferentially nucleate on magnetite accumulated at base of the magma chamber (Magnetite-α and -β). (**b**) Further ascent, growth, coalescence and accumulation of numerous magnetite-fluid-bubble pairs form an ascending magnetite-rich hypersaline suspension, which becomes Fe-rich by scavenging Fe from magma. (**c**) The fast and efficient hydraulic injection of the magnetite-rich suspension through fissures formed during the episode of collapse of the volcanic structure, forms large hydrothermal breccia bodies at depth, characterized by a matrix of an aggregate of remobilized primary magnetite (Magnetite-α and -β). The Fe-rich magmatic-hydrothermal fluid keeps ascending, crystallizing hydrothermal (Magnetite-X, -Y, and -Z) magnetite during progressive cooling until it reaches the surface, wherein it is cooled and exposed to atmospheric conditions forming Magnetite-S. (**d**) The final hydrothermal stage is represented by several veinlet types: (i) magnetite-diopside-pyrite; (ii) scapolite-magnetite-diopside (±pyrite); (iii) diopside-pyrite; (iv) magnetite-scapolite-ilmenite-pyrite (±chalcopyrite and sphalerite); (v) gypsum-magnetite-pyrite; and (vi) gypsum-pyrite that crosscut the breccia body and the volcanic host rocks. Modified from ref.^5^.

Our roots-to-surface study of the El Laco deposit reveals that El Laco is the product of a synergistic amalgamation of the following common igneous and magmatic-hydrothermal processes, which were enhanced during the evolution of a collapsing volcanic system (Fig. 4).

### Igneous magnetite segregation

Magnetite, clinopyroxene and plagioclase crystallized within the andesitic magma chamber beneath the ELVC as the dominant liquidus phases. Crystallization of these phases is consistent with the presence of disseminated magnetite in the hosting clinopyroxene-bearing andesites, as well as experimental phase equilibria studies of mafic to intermediate silicate magmas^20^. Dense magmatic phases such as magnetite are expected to undergo efficient gravitational settling in magma reservoirs, and form layers, cumulates, and/or disseminations (Fig. 4a)^21,22^. These primary magnetite grains correspond to those identified in the deep zone at El Laco (Fig. 2; Magnetite-α and Magnetite-β). Magnetite-α is characterized by well-developed ilmenite exsolution lamellae, a feature also found in accessory magnetite from andesites and dacites from El Laco and Lascar volcano^19^, respectively.

### Injection of mafic magma

The magnetite accumulation became more efficient due to multiple events of recharge, marked by the injection of pulses of mafic magma into the ELVC hydrous andesitic magma chamber. Such processes have been documented in neighboring volcanoes^23,24^, and particularly at El Laco, where observations of plagioclase-hosted melt inclusions, sieve-textured plagioclase, and chemical disequilibrium between phenocrysts and rhyodacitic groundmass from the host rock andesites, have been interpreted as reflecting mingling between felsic and mafic melts^25,26^. Open-system processes are considered an important mechanism for the upward transfer of energy and mass during magma underplating and recharge, and they can significantly contribute to both thermal rejuvenation and enrichment of transition metals and sulfur of overlying magma^27,28^. A key factor that can lead to an increase in the magnetite budget of a magma reservoir involves the injection of a crystal-bearing vapor-saturated mafic magma that intrudes into the base of an evolved crystal-rich magma chamber, wherein microlite to nanolite-sized magnetite grains and exsolved volatiles (i.e., bubbles of supercritical fluid) may be transferred, by heterogeneous nucleation processes, from the mafic intrusion into the overlying andesitic reservoir, when mixing or juxtaposition of both chemically distinct magma batches occurs (Fig. 4a)^21,22^. This mechanism has been proposed to explain the presence of magnetite-rich enclaves in arc andesites^21^. In addition, it is well known that the bubble nucleation mechanism in magmas is a predominantly heterogeneous process^29,30^ where Fe-Ti oxides are considered the only viable crystalline phases that significantly impact bubble nucleation behavior in magmas. Fe-Ti oxides provide energetically favorable sites to reduce surface energy, and decrease the supersaturation pressure required for heterogeneous nucleation^30–33^. Primary magnetite, wetted and floated by exsolved fluid bubbles within underplating and intruding mafic magma, may correspond to early-crystallized Fe-Ti oxides, fractionated from the mafic magma during its ascent through the arc crust^34–37^.

The buoyant magnetite-bubble aggregates^33^ that ascend through the mafic magma will form a magnetite-bearing, bubble-rich suspension at the interface between the intruding mafic magma and the overlying andesitic reservoir^21,38^ (Fig. 4a). The cited authors^38^ noted that the suspension layer can be transferred into the more viscous felsic magma at high gas fluxes in the mafic magma and at high viscosity ratios between both melts. In turn, if the mafic magma intrusion occurred at high injection rates, it could mix with the overlying silicic reservoir and remobilize upwards its crystal charge accumulated in the base of the magma chamber; a process consistent with numerical modeling results^39^. Hence, this mechanism would have efficiently raised the magnetite-rich cumulates from the base of the magma chamber, enhancing nucleation of exsolved fluid bubbles on remobilized magnetite crystals in suspension.

### Collapse of the volcanic edifice and remobilization of coalesced magnetite suspensions

The injection of a less evolved magma from deeper zones triggers rapid depressurization and volatile release, as commonly documented prior to explosive eruptions and collapse events^23,40–43^. At El Laco, it is well established that the ELVC morphology shows clear evidence of caldera-type collapse episodes during its volcanic history^5,26^. We argue that the collapse of the volcanic edifice played a critical role by promoting additional depressurization-derived bubble exsolution and their preferential heterogeneous nucleation on magnetite^29,30^. We propose that piston-like forces related to the collapse of the volcanic edifice promoted the efficient lateral/vertical remobilization of both a settled magnetite-rich crystal mush^41^, and coalesced magnetite-rich suspensions through pre-existing ring-shaped fissures and craters (Fig. 4b)^44^ that form a NW-trending ellipse around Pico Laco (Figs 1b and 4). The hydraulic injection of the accumulated magnetite and magnetite-suspension resulted in the formation of large volcanic and hydrothermal breccia bodies at depth below the ELVC (Figs 2, 4c and Supp. Mat. Figs S1–S3). Field observations are consistent with a Plate/Piston or Piecemeal-type collapse style^45^ induced by tectonically controlled faults, which fracture the caldera or volcanic edifice floor into numerous blocks prior to eruption^46^. Therefore, we suggest that the regional NW-trending Calama–Olacapato–El Toro structural lineament could have played an important role during the collapse stage.

### Venting of Fe-rich hydrothermal fluids

During its upward migration from the magma source, the fluid component of the magnetite-bubble suspension efficiently scavenged Cl, Fe and other metals from the silicate melt^47,48^. Igneous magnetite (Magnetite-α) grains ascended in contact with the high-temperature magmatic-hydrothermal fluid from which hydrothermal magnetite precipitated over primary magnetite during cooling and decompression (Figs 2 and 3a,b)^49–51^. High rates of magnetite nucleation and low growth rates dominated during the final ascent of the decompressing, magnetite-fluid suspension towards the surface. We argue that this Fe-rich multiphase mixture (magnetite + fluid + gas), cooled at surface temperatures, degassed extensively and sustained a rheological behavior leading to lava-like textures characteristic of the El Laco surface outcrops (Supp. Mat. Fig. S4). Evidence from numerical, experimental and empirical studies of industrial froth flotation processes shows that mineral-fluid-bubble aggregates have rheological properties similar to basaltic lava flows, i.e., both display Bingham-type behavior and exhibit similar yield stress/strength that decrease the resistance to flow^52–54^. Fluid circulation in the waning hydrothermal system or a late superimposed hydrothermal event is evidenced by the presence of sulfides-bearing magnetite-diopside-scapolite and late gypsum-magnetite-pyrite veinlets, which crosscut the main breccia bodies at depth (Fig. 4d), and the advanced argillic alteration at the surface^6^.

## Implications for iron metallogenesis in collapsing volcanic systems

The new mineralogical, geochemical and field evidence discussed here do not support a metasomatic replacement or a liquid immiscibility model for El Laco. The metasomatic replacement hypothesis^6,18,55^ is based on field observations and geochemical data of magnetite from surface samples and although our own field observations and magnetite geochemical data from surface or near surface samples (e.g., Magnetite-Z and Magnetite-S) reflect a hydrothermal origin, magnetite from the deeper roots of El Laco is unequivocally consistent with primary magmatic compositions. The depth-dependent magmatic to magmatic-hydrothermal geochemical gradients reported in this study, as well as reported magnetite Fe-O stable isotope data^56^, are consistent with magnetite crystallized both from a silicate melt and magmatic-hydrothermal fluid^56,57^, precluding complete metasomatic replacement or assimilation of ferruginous sedimentary rocks^5^. On the other hand, the models that invoke shallow emplacement of iron rich melts for El Laco formation^5,7,9,25,26,58–61^ are even more difficult to reconcile. These are based on field observations of “volcanic-like” textures in magnetite ore bodies and liquid immiscibility experiments^62,63^, which have been critically reviewed^51,64^. The experimentally constrained Δ^18^O, defined as δ^18^O_Si-rich-melt_ - δ^18^O_Fe-rich-melt_, values of 0.0 to 0.5‰^65^ at magmatic temperatures disallows liquid immiscibility to explain El Laco. Published δ^18^O values for magnetite from El Laco range from 3.5–5.5‰^58^ compared with δ^18^O values of 7 to 9‰ for silicate magmas at El Laco that would represent the conjugate Si-rich melt; the δ^18^O for magnetite from El Laco is entirely consistent with magnetite crystallized from silicate melt or high-temperature hydrothermal fluid^56^. Most importantly, recent experimental data show that during liquid immiscibility water partitions preferentially into the Si-rich melt and not into the conjugate Fe-rich melt^61^, precluding its separation and ascent from a less dense high-Si conjugate melt^26^.

In summary, we argue that the major features observed at El Laco are consistent with a formation model, wherein iron oxide bodies form as result of an optimal confluence of common subaerial volcanic processes occurring during the evolution of arc volcanoes, characterized by early magmatic and late magmatic-hydrothermal stages, marked by near-liquidus magnetite crystallization and periodic injections of crystal-bearing vapor-saturated mafic magma that trigger decompression and volatile exsolution. The efficient remobilization of magnetite-rich cumulates by the fluid-bubble-assisted flotation mechanism (e.g., heterogeneous nucleation) allows for the formation of a magnetite-fluid suspension that ascends from the magma chamber and is subsequently injected upward through collapse-related fissures and secondary craters on the flanks of a stratovolcano. This sequence of events results in venting of a multiphase mixture of magnetite crystals, silicate melt, fluid and gas that forms massive ore bodies and breccias at depth, and Fe-rich lava-like flows and pyroclastic deposits at the surface.

We drew on observations of modern arc volcanoes to invoke a magmatic-hydrothermal origin for this deposit, resulting from common igneous and hydrothermal processes operating in volcanic systems. Several occurrences of Fe oxide ores have been described in volcanic terrains around the world, although this evidence has been overlooked for decades. Intriguingly, numerous Fe oxide bodies are spatially associated with silicic volcanic rocks derived from explosive eruptions often in caldera-type volcanic environments. These occurrences include Fe deposits in Mexico^66,67^, Nevada^68^, Chile^69^, Argentina^70^, South Africa^71^, Iran^72^, and China^73^, and Fe-rich fumarolic structures in volcanic fields in Alaska^74,75^, Arizona^76^, Indonesia^77^ and Central America^78–80^. Some of these deposits display similar structural and textural features to those described at El Laco. These include dike- and vein-like Fe oxide bodies, as well as unique volcanic features such as lava-like Fe-oxide flows and scoriaceous unconsolidated Fe-oxide ash, remarkably similar to the friable ore previously described at El Laco^59,60^, and even Fe-oxide-bearing high-temperature fumarolic structures^66,74^.

Exceptional examples of magnetite precipitation from post-volcanic fumarolic activity associated with the Novarupta-Katmai eruption of 1912 were reported at the Valley of Ten Thousand Smokes in Alaska, which is considered the world’s most voluminous volcanic eruption of the 20th century, with more than ~28 km^3^ of emitted silicic volcanic material^75^. Post-volcanic high-temperature fissural fumaroles developed through ignimbrites and ash-flow sheets were reported to contain abundant loosely coherent octahedral, fine-grained magnetite^74,75^, similar to the octahedral magnetite crystals that cover the gas-escape chimney walls observed at Laco Sur and San Vicente Alto (Supp. Mat. Fig. S4). We support the notion that the occurrence of magnetite as pyroclastic friable material and as incrustations in high-temperature fumaroles indicate that the ascending magnetite-fluid suspension responsible for Fe transport and precipitation of magnetite, could undergo phase separation and fragmentation processes during ascent. We interpret that during ascent, the coalesced magnetite-fluid suspension could behave as an ascending magma prior to eruption. The magnetite-fluid suspension consists of large amounts of coalesced bubbles of compressible magmatic volatiles. Thus, owing to decompression, rapid expansion of the coalesced bubble populations plus some residual melt^60^ may trigger the break-up of the Fe-rich suspension. The individual fragments of Fe-rich material could be ejected into the atmosphere during explosive volcanic eruptions, forming pyroclast-like unconsolidated magnetite aggregates, and resulting in the rapid growth of spherulitic magnetite agglomerates. Examples include El Laco and other iron oxide-apatite deposits from Durango and Chihuahua districts in Mexico, and magnetite-rich fumaroles such as those in the Valley of Ten Thousand Smokes^59,66,74^.

Based on the global spatial and temporal distribution of iron oxide-apatite deposits^81^, it is likely that the conditions of maximum efficiency for Fe enrichment will be attained in more immature arcs such as the world-class Chilean Cretaceous Iron Belt, where the crust was thinner and mafic magma fluxes were more frequent. Nevertheless, iron deposits formed in a thick crust environment and associated with more evolved magmatism, such as the pristinely preserved El Laco in the arid high Andes, may be more frequent than previously thought and exploration efforts should be set on targeting caldera-type systems in active and fossil magmatic arcs.

## Samples and Methods

Our study combined field observations and drill core logging with detailed micro-textural observations and elemental micro-analysis of magnetite. We retrieved 159 samples from seven drill cores at several depths from Laco Norte, Laco Sur, Extención Laco Sur and Pasos Blancos (Fig. 1b). In addition, 39 samples were obtained from 5 outcropping massive magnetite ore bodies (Laco Norte, Laco Sur, San Vicente Alto, Rodados Negros and Cristales Grandes). Drilling was performed by Compañía Minera del Pacífico (CMP) during an exploration program between 2007–2010.

In order to identify the textural variability of the magnetite grains from depth to surface, forty seven representative samples were inspected and imaged by using a JEOL 7800FLV field emission-scanning electron microscope (FE-SEM) at the University of Michigan and a Model FEI Quanta 250 SEM at Universidad de Chile. Additionally, to comprehensively characterize the chemical variability of the different magnetite types throughout the deposit and at depth, 1912 electron probe microanalyses (EPMA) were obtained by using a Cameca SX-100 at the University of Michigan, USA (Electron Microbeam Analysis Laboratory, EMAL). Magnesium, Al, Si, Ca, P, Ti, V, Cr, Mn and Fe were analyzed in magnetite grains, and interference corrections were carried out for Ti concentrations since V Kβ affects the Ti Kα signal. The operating conditions employed were an accelerating voltage of 20 keV and a focused beam to avoid measuring inclusions or ilmenite exsolution lamellae in magnetite. The beam current was set to 30 nA. A counting time of 20 s was used for Fe, while counting times of 100 s (Ca, Cr, Mn), 110 s (Si, P, Mg, Al) and 120 s (Ti, V) were used for minor and trace elements. A variety of natural and synthetic oxides and silicates were used as primary standards for each element. The standards, as well as the EPMA analytical conditions used are summarized in Supplementary Material (Supplementary Table 1). The database of magnetite mineral chemistry is provided as Excel file in Supplementary Material (Appendix_1). The measurements that yield values below detection limit are listed in Appendix _1 as “b.d”. However, to build the [Al + Mn] versus [Ti + V] diagram, such below detection limit values, were replaced by the half of the corresponding detection limits.

## Electronic supplementary material

Supplementary Information

Appendix 1

## Data Availability

All data generated or analyzed during this study are included in this published article (and its Supplementary Material files).
