# Supplementary material for: Formation of massive iron deposits linked to explosive volcanic eruptions
Source: Sci Rep. 2018 Oct 5;8:14855. doi: 10.1038/s41598-018-33206-3 (PMC6173703; doi:10.1038/s41598-018-33206-3)
Supplement: Supplementary file 1 — Supplementary Information [file 41598_2018_33206_MOESM1_ESM.pdf]

Supplementary Material for:

Formation of massive iron deposits linked to explosive volcanic eruptions

J. Tomás Ovalle[1,*], Nikita L. La Cruz[2], Martin Reich[1], Fernando Barra[1], Adam C. Simon[2], Brian Konecke[2], María A. Rodriguez-Mustafa[2], Artur P. Deditius[3], Tristan Childress[2], Diego Morata[1]

correspondence to: joseovalle@ug.uchile.cl

This PDF file includes:

Supplementary Table 1
Supplementary Figure S1
Supplementary Figure S2
Supplementary Figure S3
Supplementary Figure S4

Other Supplementary Material for this manuscript includes the following:

Appendix_1 (Excel file)

Supplementary Table 1: Summary of EPMA conditions for magnetite analysis.

Element/line	Crystal	Standard	Counting time (s)	MDL (wt.%)
**Fe Kα**	LLIF	USMN Magnetite	20	0.02
**Ti Kα**	PET	USMN Ilmenite	120	0.01
**V Kα**	LLIF	V_2O_5	120	0.01
**Cr Kα**	LLIF	Cr_2O_3	100	0.01
**Al Kα**	TAP	Jadeite	100	0.01
**Mn Kα**	LLIF	Rhodonite (BHRH)	100	0.01 - 0.03
**Si Kα**	LTAP	Wollastonite	100	0.01
**Mg Kα**	TAP	Geikielite (GEIK)	100	0.01 - 0.02
**Ca Kα**	PET	Wollastonite	100	0.01
**P Kα**	LTAP	BaCl	100	0.01 - 0.02

MDL: mean detection limit.

Supplementary Figure S1:

This figure is an expanded version of Figure 2 in the main text, where pictures of hand specimens are shown for each sample (Drill core LCN-0944)

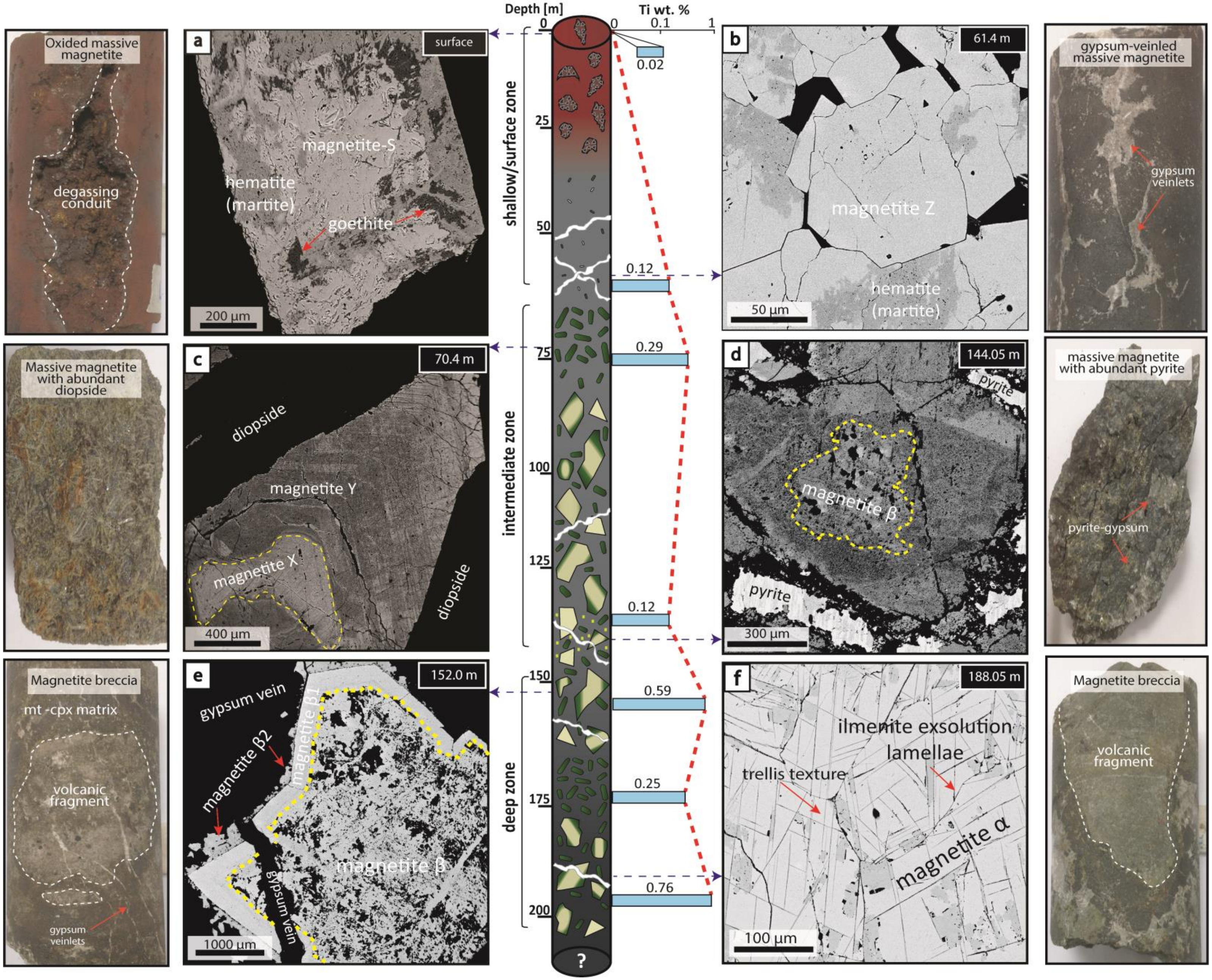

Supplementary Figure S2:

Schematic illustration of a representative drill core from Laco Sur showing the morphology and lithological variations of the magnetite ore bodies (drill core LCO-0721A). BSE images of magnetite textural types are shown (center), as well as pictures of hand specimens taken at different depths (left): **(a)** Euhedral magnetite grain showing inclusion-rich cores surrounded by pristine magnetite rims. Hematite (± goethite) are observed along the grain rims. **(b)** Coarse magnetite grain containing inclusion-rich cores surrounded by more pristine magnetite rims. **(c)** Relatively pristine magnetite from the breccia matrix, containing inclusions of scapolite and clinopyroxene. The andesitic fragments are pervasively replaced by fine-grained scapolite. **(d)** Magnetite from late veins exhibiting crystallographically-controlled, alternating inclusion-rich and inclusion-poor zones (blue arrows). mt: magnetite, cpx: clinopyroxene, scp: scapolite.

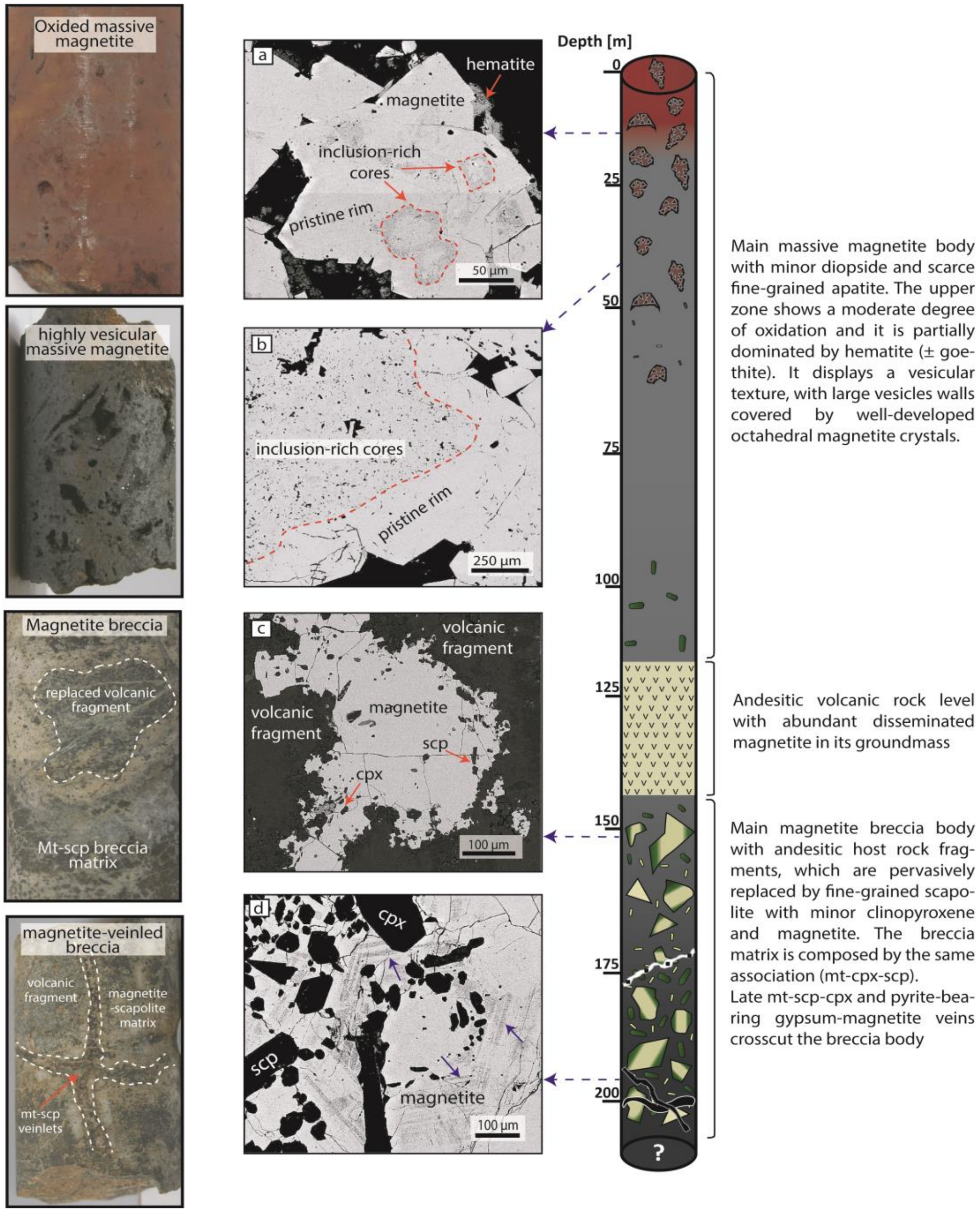

Supplementary Figure S3:

Schematic illustration of a representative drill core from Laco Sur showing the morphology and lithological variations of the ore bodies (drill core LCO-0715). BSE images of magnetite textural types are shown (center), as well as pictures of hand specimens taken at different depths (left): **(a)** Euhedral coarse-grained magnetite grain exhibiting resorbed inclusion-rich cores surrounded by magnetite rims with abundant nano-sized inclusions. **(b)** Magnetite grain from the breccia matrix containing abundant scapolite and clinopyroxene. **(c)** Ti-rich magnetite from the breccia matrix, showing well-developed ilmenite exsolution lamellas, which exhibit both trellis and sandwich textures.

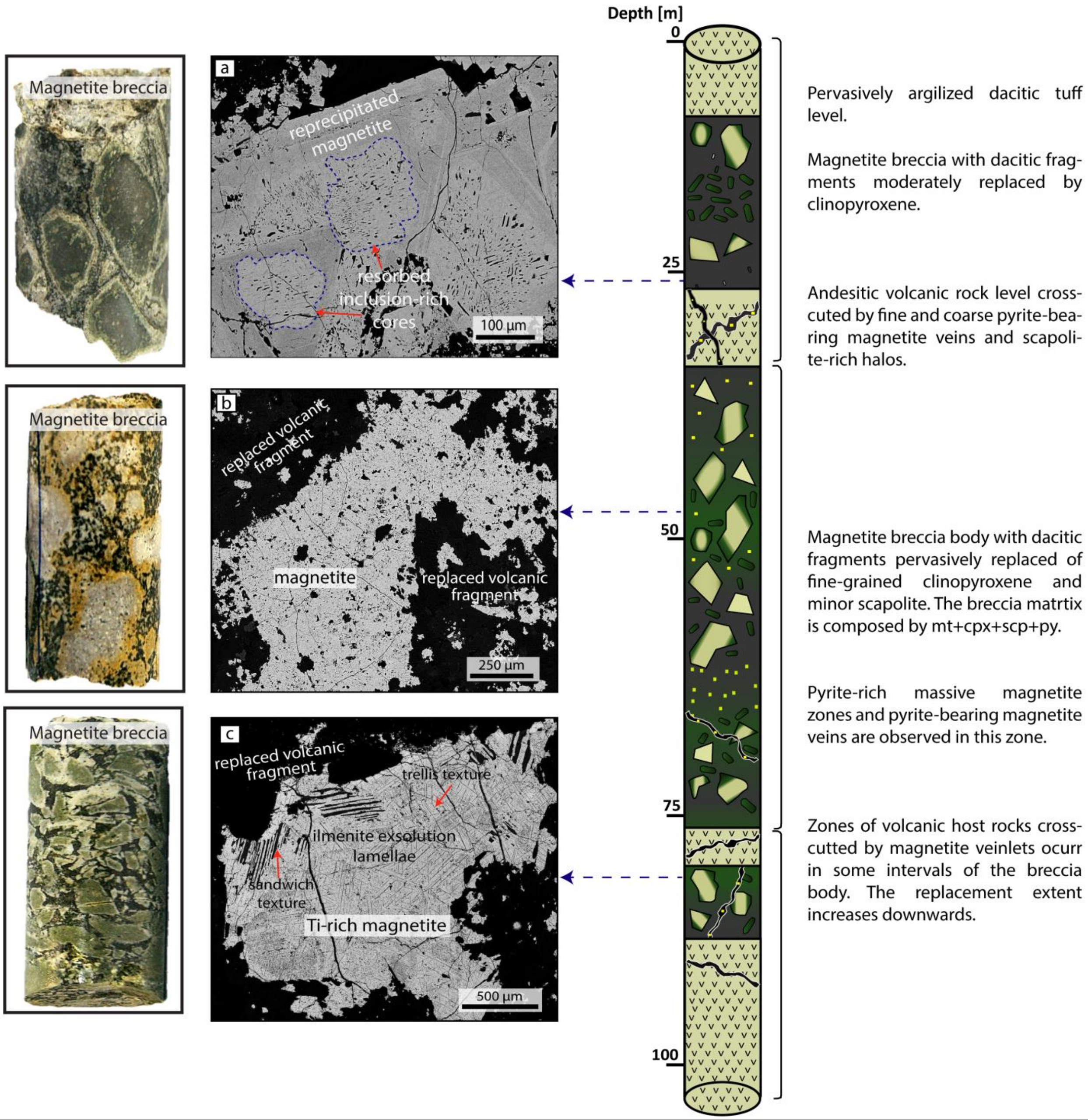

<u>Supplementary Figure S4</u>:

Pictures of outcrops and hand samples from the El Laco deposit: **(a-c)** Outcrop of the Laco Norte ore body. Note the highly vesicular texture. **(d-e)** Outcrop of the San Vicente Alto ore body. Note the degassing structures including vesicles, pores and rounded structures similar to bubble aggregates (d), and subvertical degassing conduits (e). **(f)** Massive magnetite outcrop from Laco Norte showing pahoehoe-type flow structures.

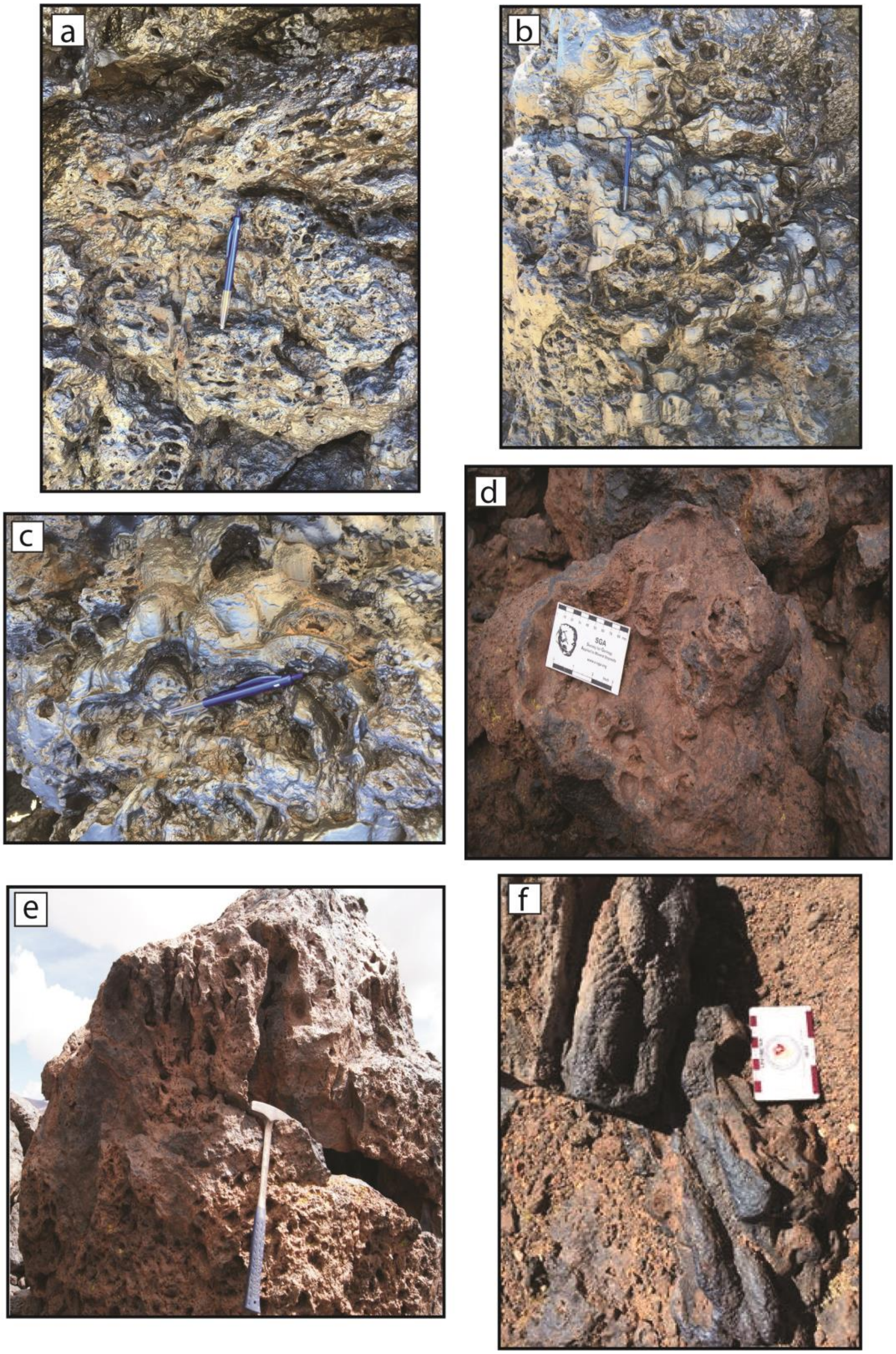

Apendix_1 (separate Excel file)

All EPM analysis of magnetite for studied ore bodies.
